# Evidence of Hyperacetylation of Mitochondrial Regulatory Proteins in Left Ventricular Myocardium of Dogs with Chronic Heart Failure

**DOI:** 10.3390/ijms26083856

**Published:** 2025-04-18

**Authors:** Ramesh C. Gupta, Kristina Szekely, Kefei Zhang, David E. Lanfear, Hani N. Sabbah

**Affiliations:** Department of Medicine, Division of Cardiovascular Medicine, Henry Ford Heart & Vascular Institute, Henry Ford Hospital, Detroit, MI 48202, USA; rgupta1@hfhs.org (R.C.G.); kszekel1@hfhs.org (K.S.); kzhang1@hfhs.org (K.Z.); dlanfea1@hfhs.org (D.E.L.)

**Keywords:** canine, left heart failure, mitochondria, hyperacetylation, protein levels

## Abstract

Increased acetylation or “hyperacetylation” of mitochondrial (MITO) proteins can lead to abnormalities of the electron transport chain (ETC) and oxidative phosphorylation. In this study we examined the levels of proteins that regulate acetylation. Studies were performed in isolated MITO fractions from left ventricular (LV) myocardium of seven healthy normal (NL) dogs and seven dogs with coronary microembolization-induced heart failure (HF, LV ejection fraction ~35%). Protein levels of drivers of hyperacetylation, namely sirtuin-3 (Sirt-3), a MITO deacetylase, and CD38, a regulator of nicotinamide adenine dinucleotide (NAD^+^), were measured by Western blotting, and the bands were quantified in densitometric units (du). To assess MITO function, MITO components directly influenced by a hyperacetylation state, namely the protein level of cytophillin-D (CyPD), a regulator of MITO permeability transition pore and MITO Complex-I activity, were also measured. Protein level of Sirt-3 and amount of NAD^+^ were decreased in HF compared to NL dogs. Protein levels of CD38 and CyPD were increased in HF compared to NL dogs. Complex-I activity was decreased in HF compared to NL dogs. The results support the existence of a protein hyperacetylation state in mitochondria of failing LV myocardium compared to NL. This abnormality can contribute to MITO dysfunction as evidenced by reduced Complex-I activity and opening of MITO permeability pores.

## 1. Introduction

Heart failure (HF) is a progressive disorder and a major public health issue worldwide, with an estimated 57 million people affected as of 2021. The prevalence of HF is expected to increase by nearly 46% by 2030 due to an increase in the aging population, improved survival after HF diagnosis, and the rise of risk factors such as coronary artery disease, hypertension, obesity, and diabetes. Despite the availability of effective therapies, the 5-year HF mortality rate in the United States remains unacceptably high at nearly 50%. The heart is a high energy-consuming organ, and mitochondria within cardiomyocytes serve as the powerhouse for the energy needed to meet the demands of cardiac contraction and relaxation. Unfortunately, mitochondria of the failing heart are dysfunctional; a maladaptation that contributes to progressive worsening of the HF state [1,2,3,4]. Novel therapies that target MITO in HF carry considerable promise. However, previous work focusing on improving mitochondrial (MITO) energy production and reducing reactive oxygen species (ROS) in HF has yielded limited clinical success [4,5].

In recent years, protein lysine acetylation has emerged as an important mechanism linking cellular metabolism to cellular signaling [6]. The level of protein acetylation reflects the balance of acetylation and deacetylation. While the former is dependent on the abundance of acetyl-CoA and/or the activity of acetyltransferase, the latter is determined by the deacetylase activity driven by sirtuin levels in the MITO, mainly sirtuin-3 (Sirt-3) [7,8]. Sirtuin-3 plays a crucial role in regulating MITO function, metabolism, and stress responses, including energy generation, oxidative stress, and cell death. Recent studies suggest that acetyl-CoA levels are increased in the failing human heart [9,10], an adaptation that can promote hyperacetylation of MITO nonhistone proteins [11]. The latter can cause abnormalities in the MITO electron transport chain (ETC) complexes, leading to reduced ATP production and a potential increase in the formation of reactive oxygen species (ROS). Hyperacetylation can also cause increased levels of cyclophilin-D (CyPD) within MITO, leading to the opening of mitochondrial permeability transition pores (mPTP) [12]. Cyclophilin D is a peptidyl-prolyl isomerase that also plays a crucial role in MITO function and cell death, particularly in ischemia/reperfusion injury, HF, and neurodegenerative diseases. Acetylation levels in mitochondria can also be regulated by changes in the expression of the glycoprotein CD38 through its action on nicotinamide adenine dinucleotide (NAD^+^). As such, increased or decreased expression of CD38 in mitochondria can result in a decrease or an increase of NAD^+^, respectively, leading to acetylation or deacetylation of MITO proteins [13,14]. CD38 is a multifunctional ectoenzyme and transmembrane glycoprotein involved in various cellular processes, including calcium signaling, immune cell activation, and the metabolism of NAD^+^.

In this study, we tested the hypothesis that hyperacetylation of nonhistone proteins occurs in mitochondria of the failing left ventricle (LV) of dogs with HF, as evidenced by altered protein levels of sirt-3, CD38, and CyPD. We also assessed the integrity of the ETC through measurement of MITO Complex-I activity. The above hypothesis was tested in an established model of human HF in the dog described more fully in the methods section. All animal models of human disease are limited but nonetheless very useful in exploring hypotheses designed to better understand human disease pathophysiology.

## 2. Results

### 2.1. NAD^+^ and NADH Protein Levels

**Figure 1:**
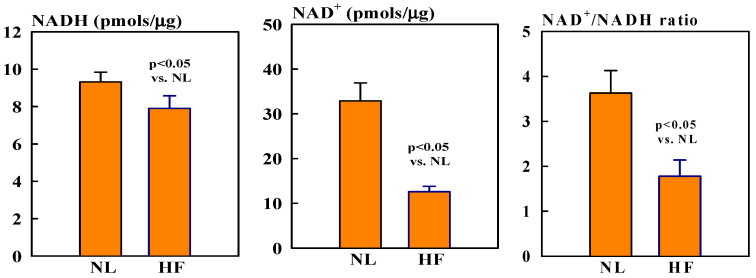
Bar graphs (mean ± SEM) of levels of NAD (**left**), NADH (**middle**), and NAD^+^/NADH ratio (**right**) in isolated mitochondrial fractions of left ventricular (LV) myocardium of seven healthy normal (NL) dogs and seven dogs with heart failure (HF).

Mitochondrial NAD^+^ levels were ~60% lower in HF dogs compared to healthy normal (NL) dogs (12.6 ± 1.2 vs. 32.9 ± 4.0, pmols/µg, *p* < 0.05). In contrast, NADH levels were only slightly, but not significantly, lower in HF dogs compared to NL dogs (7.9 ± 0.7 vs. 9.3 ± 0.5 pmols/µg). The ratio NAD^+^/NADH was nearly 118% lower in HF dogs compared to NL dogs (1.8 ± 0.4 vs. 3.6 ± 0.5, *p* < 0.05) (Figure 1).

### 2.2. Mitochondrial Sirt3, CD38, CyPD, and Porin Protein Levels

**Figure 2:**
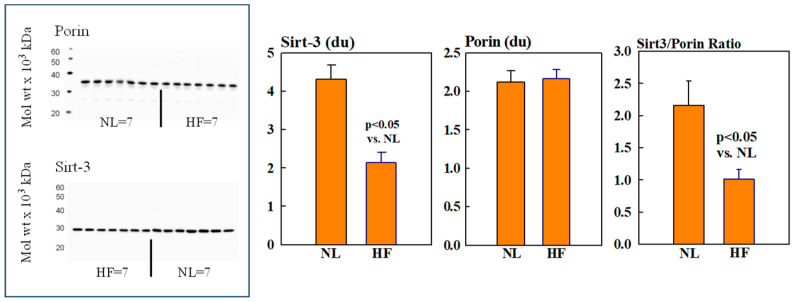
(**Left**): Western blots of sirtuin-3 (Sirt3 and Porin) in mitochondrial fractions of seven healthy normal dogs (NL) and seven dogs with heart failure (HF). (**Right**): Bar graphs (mean ± SEM) depicting protein levels of Sirt3 (**left**), porin (**middle**), and the ratio of Sirt3/porin (**right**) in mitochondrial fractions of LV myocardium of seven healthy NL dogs and seven dogs with HF.

**Figure 3:**
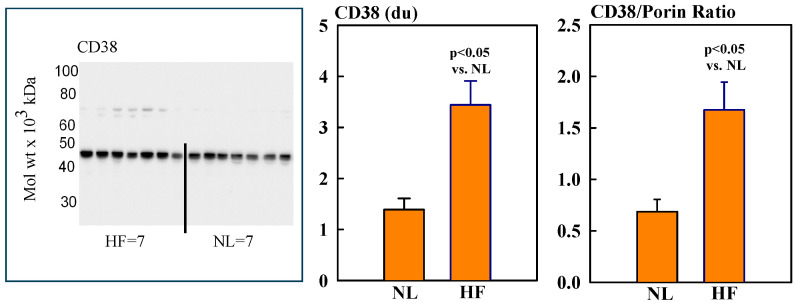
(**Left**) Western blot of CD38 in mitochondrial fractions of seven healthy normal dogs (NL) and seven dogs with LV heart failure (HF). (**Right**) Bar graphs (mean ± SEM) depicting protein levels of CD38 and the ratio of CD38/porin in mitochondrial fractions of LV myocardium of seven healthy NL dogs and seven dogs with HF.

**Figure 4:**
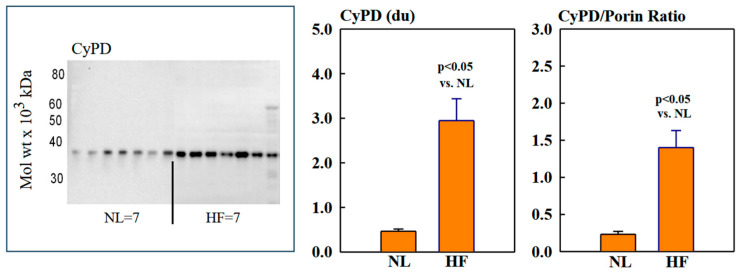
(**Left**) Western blot of cyclophilin-D (CyPD) in mitochondrial fractions of seven normal dogs (NL) and seven dogs with heart failure (HF). (**Right**) Bar graphs (mean ± SEM) depicting protein levels of CyPD and the ratio of CyPD/porin in mitochondrial fractions of LV myocardium of seven healthy NL dogs and seven dogs with HF.

Mitochondrial protein levels of porin, used as an internal control, were essentially the same among NL and HF dogs (2.12 ± 0.15 vs. 2.16 ± 0.12 du) (Figure 2). Protein levels of MITO Sirt3 were lower in HF dogs compared to NL dogs (2.14 ± 0.27 vs. 4.31 ± 0.36 du). The ratio Sirt3/porin was significantly lower in HF dogs compared to NL dogs (1.01 ± 0.15 vs. 2.14 ± 0.27 du, (*p* < 0.05) (Figure 2). Mitochondrial CD38 protein levels were significantly higher in HF dogs compared to NL dogs (3.44 ± 0.47 vs. 1.39 ± 0.22 du. The ratio of CD38/porin was 0.77 ± 0.03 in NL dogs and was significantly increased to 1.69 ± 0.12 in HF dogs (*p* < 0.05) (Figure 3). Mitochondrial CyPD protein levels were increased in HF dogs compared to NL dogs (2.95 ± 0.49 vs. 0.47 ± 0.10 du). The ratio of CyPD/porin was 0.23 ± 0.04 in NL dogs and was significantly increased to 1.40 ± 0.23 in HF dogs (*p* < 0.05) (Figure 4).

### 2.3. Complex-1 Activity

**Figure 5:**
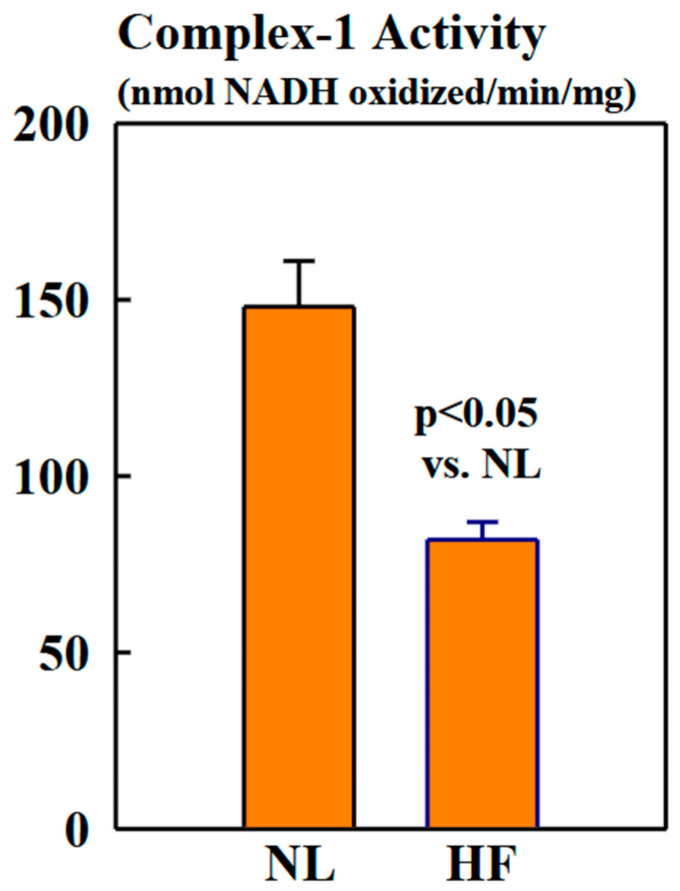
Bar graphs (mean ± SEM) depicting Complex-1 activity assayed in isolated mitochondrial fractions of LV myocardium of six healthy NL dogs and seven dogs with HF.

Complex-1 activity was significantly lower in mitochondria of HF dogs compared to NL dogs (82 ± 5 vs. 148 ± 13 nmols NADH oxidized/min/mg, *p* < 0.05) (Figure 5).

## 3. Discussion

Results of the present study indicate that constituent mitochondria of cardiomyocytes of LV myocardium of dogs with HF manifest decreased NAD^+^/NADH, reduced Sirt-3 protein levels, increased CD38 and CyPD protein levels, and reduced Complex-1 activity compared to mitochondria of LV myocardium of NL dogs. These findings, when viewed in concert, provide compelling evidence of the existence of a hyperacetylation state in mitochondria of failing LV myocardium. This increased protein acetylation accounts, in part, for previously documented structural and functional abnormalities of mitochondria of the failing left ventricle [15,16,17,18].

Aging and acute ischemic events are associated with declines in the heart’s NAD^+^ levels, linked to NADases such as CD38, which degrade NAD^+^ during its catalytic process [13]. Previous studies have shown that CD38 knockout mice have higher NAD^+^ levels that protect against obesity and metabolic syndrome [14,19]. Treatment of obese mice with CD38 inhibitors has been shown to increase intracellular NAD^+^ levels and improve several aspects of glucose and lipid homeostasis [19]. However, the role of CD38 in the decline of NAD^+^ in mitochondria of the failing LV myocardium in HF is not known. In the present study, we observed an increase in CD38 associated with decreased NAD^+^ levels. A mouse model of primary MITO dysfunction caused by cardiac-specific deletion of the Complex-1 protein subunit NADH:Ubiquinone Oxidoreductase Subunit S4 (Ndufs4) resulted in a decrease in the NAD^+^/NADH ratio, leading to MITO protein hyperacetylation and increased susceptibility to stress [20,21]. In the same study, the authors suggested that restoring the NAD^+^/NADH ratio by genetic and pharmacological approaches may represent a potentially effective therapy for treating HF, a condition associated with a reduced NAD^+^/NADH ratio.

It is well known that a decrease in the ratio of NAD^+^/NADH results in reduced Sirt3 activity, an enzyme having deacetylase activity. Therefore, the reduction in Sirt3 protein levels observed in the present study in the failing heart supports an increased protein acetylation state. Increased acetylation of MITO proteins can give rise to increased CyPD protein levels observed in the present study. CyPD, through its covalent modifications, particularly acetylation, is a modulator of mPTP opening [22,23,24,25]. Increased opening of mPTP can lead to the release of cytochrome-c from mitochondria, leading to programmed cell death, or apoptosis. The exact mechanism by which CyPD causes opening of the mPTP is not well known, but it may be due to binding of CyPD to inner MITO membrane components, such as adenine nucleotide translocator (ANT) [22] and ATP synthase [26], that, in turn, can trigger opening of mPTP [27]. We previously showed increased opening of mPTP in dogs with coronary microembolization-induced HF compared to healthy NL dogs [15].

Mitochondrial Complex-1 is composed of 45 subunits. It transfers electrons from NADH to coenzyme Q_10_ and expels 4H^+^ into the intermembrane space, thereby contributing to the mitochondrial membrane potential (Ψ_m_) [28]. Complex-1 is one of the most important contributors to the formation of reactive oxygen species production [21], a source of lipid peroxidation. Increased acetylation of two subunits of Complex-1, NADH:Ubiquinone Oxidoreductase Subunits NDUFA9 and NDUFA11, has been reported to cause reduction in Complex-1 activity [29]. In the present study, we observed reduced Complex-1 activity in mitochondria of LV myocardium of dogs with HF compared to NL dogs, indicating increased acetylation.

## 4. Materials and Methods

### 4.1. Procurement of LV Tissue

The present study was performed using frozen LV tissue samples from our dog tissue banks. The tissue samples used were from 7 healthy male mongrel dogs and 7 male dogs with left ventricular HF induced by multiple intracoronary microembolizations [15,30]. Upon arrival at our institution, dogs were maintained in our fully accredited bioresources facilities in large individual pens. Dogs underwent a 2-week period of acclimation before entry into the study. The total stay in our bioresources facility ranged between 7 and 9 months. All dogs underwent a complete examination by the attending veterinarian along with complete blood work. In addition, all dogs underwent two-dimensional echocardiographic studies to assess cardiac function. All dogs were deemed within normal limits before being entered into the study. The canine model of HF used in this study replicates many of the sequelae of left-sided HF seen in humans and is devoid of right-sided failure. The dog coronary microembolization model is based on injecting microspheres sub-selectively into the left anterior descending coronary artery and circumflex coronary artery while avoiding the right coronary artery [15,30]. The latex microspheres (approximately 90 µm in diameter) are trapped in the coronary microcirculation, leading to ischemia and small infarctions, i.e., loss of viable myocardium. After multiple coronary microembolization sessions, the total infarcted myocardium volume in these dogs can range between 20% and 35%, leading to a reduction in LV ejection fraction and to LV dilation indicative of LV failure [15,30]. The reduction in LV ejection fraction is always accompanied by a reduction in LV stroke volume and cardiac output and an increase in peripheral vascular resistance, all of which are hallmarks of left-sided heart failure in humans [15,30]. Because the right coronary artery is not embolized, dogs do not develop right-sided failure, i.e., right ventricular failure.

At the time of study, the dogs were 12 to 18 months of age, and their weight ranged between 21 and 26 kg. To induce HF, dogs underwent intracoronary microembolizations with polystyrene latex microspheres (77 to 102 gm in diameter) performed during sequential cardiac catheterizations under general anesthesia and sterile conditions performed 1 to 2 weeks apart. Coronary embolizations were discontinued when LV ejection fraction, assessed by echocardiography and ventriculography, was ~35% [15,30]. Thereafter, dogs were maintained for 4–6 months before tissue was harvested. None of the dogs were treated with cardioactive drugs. All echocardiographic procedures were performed by a trained sonographer (author KZ), and all hemodynamic and cardiac catheterization procedures were performed by qualified hemodynamic and animal cardiac catheterization experts (authors HNS and KZ).

At the time of tissue harvesting, dogs were anesthetized, the chest and pericardium were opened, and the heart was rapidly removed and immersed in ice-cold cardioplegia solution. At the time of tissue harvesting, dogs showed no signs of cardiac decompensation, namely, pulmonary congestion/edema, pericardial effusion, or ascites. Transmural tissue sections from the LV free wall were frozen in liquid nitrogen and stored at −70 °C until used. The study was approved by the Henry Ford Health System Institutional Animal Care and Use Committee (IACUC) and conformed to the National Institute of Health Guide and Care for Use of Laboratory Animals (National Institutes of Health publication No. 85-23). The IACUC approval codes for dogs used in this study were 1144 and 1171. The approval dates were 5 October 2011 and 8 February 2012, respectively. Animals were euthanized, and cardiac tissue was collected and stored at −70 °C in our tissue banks between February 2012 and July 2012. Dogs were approved by IACUC for investigations unrelated to the present study. The present study was conducted using LV tissue samples from these dogs that were stored at −70 °C in tissue banks. Use of banked tissue does not require additional IACUC approval. All biochemical assays reported in the present study using banked LV tissue samples were performed between the years 2021 and 2023.

### 4.2. Isolation of Mitochondria

Mitochondrial fractions were isolated from thawed LV tissue by differential centrifugation as previously described [31]. Briefly, approximately 2 g of frozen LV tissue was thawed and subsequently quickly minced using scissors into small pieces in 20 mL of ice-cold Buffer-A (100 mM KCl, 50 mM 3-[N-Morpholino]-propanesulfonic acid (MOPS), 5 mM MgSO_4_, 0.5 mM EGTA, 1 mM ATP, pH 7.4). The scissor-minced tissue was then transferred into a glass Potter-Elvehjem tube containing 20 mL of Buffer-B (Buffer-A + 1 mg/mL BSA). Then, with three strokes of a Teflon pestle, the tissue was gently homogenized using a polytron (VWR, Radnor, PA, USA) for 3 times × 3 s at setting 3. The resulting homogenate was centrifuged (Beckman J2HS centrifuge with JA20 rotor, Indianapolis, IN, USA) at 584 g for 10 min. After centrifugation, the supernatant containing mostly subcellular mitochondria, located beneath the sarcolemma, was collected in a separate tube and then centrifuged for 10 min at 3000× *g*. The pellet was resuspended in 10 mL of KME buffer (100 mM KCl, 50 mM MOPS, and 0.5 mM EGTA, pH 7.4) [31] and recentrifuged at 9000× *g* for 10 min. The final sediment was resuspended in 0.6 mL of KME buffer and labeled as mitochondria, which was finally aliquoted (100 µL/aliquot) followed by quickly freezing in liquid nitrogen and then stored at −70 °C. All the reagents used in homogenizing LV tissue were obtained from Sigma-Aldrich (St. Louis, MO, USA). Protein concentration of MITO fractions was determined by using a DC protein assay kit (Bio-Rad, Hercules, CA, USA), which is a colorimetric assay that measures protein concentrations using a modified Lowry method. The DC assay is compatible with detergents and therefore widely used for accurate quantification of proteins in mitochondrial extracts using Bovine Serum Albumin as a standard. It involves the reaction of peptide bonds with copper ions under alkaline conditions, forming a complex that reduces the Folin–Ciocalteau reagent, resulting in a blue color. The intensity of this color, measured spectrophotometrically, is proportional to protein con-centration.

### 4.3. Determination of Mitochondrial NAD^+^ and NADH Levels

Mitochondrial NAD^+^ and NADH levels were determined using an NAD^+^/NADH quantitation kit (abcam, Cambridge, MA, USA). Briefly, NAD^+^ and NADH were extracted from approximately 500 μg of mitochondrial fraction using specific buffers. After incubation at room temperature for 15 min, an aliquot of 25 μL was added to 75 μL of NAD cycling buffer in a microplate, and OD was recorded at 340 nm using a Biotek Synergy microplate reader (Agilent, Winooski, VA, USA). Total NADt (total NAD^+^ and NADH) and NADH were measured, and then the level of NAD^+^ was calculated by subtracting NADH from NADt and expressed as pmols/µg protein.

### 4.4. Complex-1 Activity Assay

The activity of MITO Complex-1 was assayed spectrophotometrically using a Biotek Synergy Microplate Reader (Agilent, Winooski, VA, USA), as previously described [21], by monitoring the oxidation of NADH (0.25 mM) at 340 nm at 30 °C in the assay buffer containing 62.5 μM ubiquinone, 0.25% BSA, antimycin A (2 μg/mL), and mitochondria in the absence and presence of rotenone (10 μg/mL). Considering molecular absorptivity of NADH as 6.22, Complex-1 activity was calculated as the rotenone-sensitive NADH:ubiquinone oxidoreductase activity and expressed as nmoles/min per mg protein. All the reagents used in the assay were obtained from Sigma-Aldrich (Ann Arbor, MI, USA).

### 4.5. Western Immunoblotting

Protein levels of Sirt3, CyPD, CD38, and porin were determined in isolated mitochondria using their specific, commercially available antibody and employing Western blotting coupled with chemiluminescence, a powerful technique for detecting specific proteins in a complex mixture.

Briefly, mitochondria were isolated from LV tissue, and protein concentration was determined using a DC protein assay kit (Bio-Rad, Hercules, CA, USA) as previously described [32]. SDS extracts of isolated mitochondrial fractions were prepared, and then approximately 2–10 μg of protein was subjected to Western blotting as described previously [32]. Porin was used as an internal control for normalization. Porin is a protein that is present in mitochondria of cardiomyocytes. It is well known that porin levels are not altered in HF, making this protein ideal for normalization to avoid discrepancies that may arise from sample-to-sample variations. After separating proteins on 4–20% SDS-PAGE (Bio-Rad, Hercules, CA, USA) and transferring to PVDF membrane (Bio-Rad, Hercules, CA, USA) using Transblot Turbo (Bio-Rad, Hercules, CA, USA). Blots were then blocked with Superblock T20 blocking buffer (Thermo Scientific, Waltham, MA, USA) for 2 h at room temperature, treated with specific primary antibodies for approximately 18 h at 4 °C, followed by the corresponding secondary antibodies coupled with horseradish peroxidase for 2 h at room temperature. The bands on the PVDF membrane were developed by ECL color-developing reagents for 10–15 s at room temperature, according to the supplier (Amersham, UK). The band intensity was quantified using an imaging densitometer (ChemiDoc^TM^ Touch System, Bio-Rad) and expressed as densitometric units (du). In all cases, it was ensured that the antibody was present in excess over the antigen and the density of each protein band was in the linear scale. Primary antibodies used were Sirt3 rabbit mAb (Cell Signaling Technology, Danvers, MA, USA) with reactivity to human, canine, rabbit, and monkey; CyPD (rabbit pAb, Enzo Life Sciences, Inc., Farmingdale, NY, USA) with reactivity to mouse, canine, and human; CD38 (mouse mAb, Santa Cruz Biotechnology, Santa Cruz, CA, USA) with reactivity to human; and porin with reactivity to human (rabbit pAb, Abcam, Cambridge, UK). The CD38 was not specific to canines. However, we observed clear, single-band cross-reactivity with canine heart samples by Western blotting at a molecular weight of 45 kDa, consistent with that expected for CD38.

### 4.6. Statistical Analysis

All data were examined for non-normal distribution prior to selecting the statistical test. Statistical comparisons between NL and HF dogs for all measures were made using a t-statistic for two means with significance set at *p* < 0.05 (Primer of Biostatistics, 7th Edition, McGraw Hill Publishing, New York City, NY, USA). The data showed normal distribution. All data are expressed as means ± standard error of the mean ± SEM.

## 5. Conclusions

Results of the present study indicate that mitochondria of LV myocardium of dogs with HF manifest decreased NAD^+^/NADH, reduced Sirt-3 protein level, increased CD38 and CyPD protein levels, and reduced Complex-1 activity compared to mitochondria of LV myocardium of healthy NL dogs. These findings, when viewed in concert, provide strong evidence for the existence of a hyperacetylation state in mitochondria of failing LV myocardium of dogs. We speculate that therapeutic maneuvers that can potentially reverse or ameliorate the above adverse cascade of events may represent a viable approach to treating abnormalities of mitochondria in HF. Mitochondrial proteins from the LV of dogs with HF manifest increased levels of acetylation. The increased protein acetylation identified in this study is probably caused by decreased Sirt3 and increased CyPD and CD38 protein levels. Reduced Complex-1 activity and decreased NAD^+^/NADH ratio in MITO of dogs with HF also play a role in the increased protein acetylation. These defects in the MITO of the LV myocardium of failing dog hearts represent an adverse maladaptation capable of increasing acetylation of CyPD and thus increasing mPTP openings, which are detrimental to heart function.

## Data Availability

Data will be made available upon request.
